# Role of Respiratory Viruses in Severe Acute Respiratory Failure

**DOI:** 10.3390/jcm14093175

**Published:** 2025-05-03

**Authors:** David Mokrani, Jean-François Timsit

**Affiliations:** 1 Infectious and Intensive Care Unit, Centre Hospitalier Universitaire Bichat-Claude Bernard, Assistance Publique-Hôpitaux de Paris, 75018 Paris, France; david.mokrani@aphp.fr; 2 Infection Antimicrobials Modelling Evolution (IAME), Mixt Research Unit (UMR) 1137, INSERM, Université Paris-Cité, 75018 Paris, France

**Keywords:** ARDS, pneumonia, acute respiratory failure, viruses, mPCR, influenzae, pneumoviridae, respiratory syncytial virus, metapneumovirus, baloxavir, oseltamivir, emergent infections

## Abstract

Respiratory viruses are widespread in the community, affecting both the upper and lower respiratory tract. This review provides an updated synthesis of the epidemiology, pathophysiology, clinical impact, and management of severe respiratory viral infections in critically ill patients, with a focus on immunocompetent adults. The clinical presentation is typically nonspecific, making etiological diagnosis challenging. This limitation has been mitigated by the advent of molecular diagnostics—particularly multiplex PCR (mPCR)—which has not only improved pathogen identification at the bedside but also significantly reshaped our understanding of the epidemiology of respiratory viral infections. Routine mPCR testing has revealed that respiratory viruses are implicated in 30–40% of community-acquired pneumonia hospitalizations and are a frequent trigger of acute decompensations in patients with chronic comorbidities. While some viruses follow seasonal patterns, others circulate year-round. Influenza viruses and Pneumoviridae, including respiratory syncytial virus and human metapneumovirus, remain the principal viral pathogens associated with severe outcomes, particularly acute respiratory failure and mortality. Bacterial co-infections are also common and substantially increase both morbidity and mortality. Despite the growing contribution of respiratory viruses to the burden of critical illness, effective antiviral therapies remain limited. Neuraminidase inhibitors remain the cornerstone of treatment for severe influenza, whereas therapeutic options for other respiratory viruses are largely lacking. Optimizing early diagnosis, refining antiviral strategies, and systematically addressing bacterial co-infections are critical to improving outcomes in patients with severe viral pneumonia.

## 1. Introduction

With the increasing use of molecular assays, the detection of viral pathogens in critically ill adults with respiratory illnesses has become more common. The reported prevalence rates range from 17% to 53%, depending on factors such as the study design, sample type, illness duration, and assay techniques. Viruses most frequently identified in patients with severe respiratory illnesses include influenza A and B, picornaviruses (like rhinovirus and enterovirus, e.g., enterovirus D68), human coronaviruses (229E, NL63, OC43, and HKU1), respiratory syncytial virus (RSV), human metapneumovirus (hMPV), parainfluenza virus, and adenovirus. In addition, emerging zoonotic coronaviruses—such as those causing Severe Acute Respiratory Syndrome (SARS), Middle East Respiratory Syndrome (MERS), and COVID-19—continue to be identified. However, attributing clinical illness to a specific viral pathogen remains challenging. Some viruses, such as picornaviruses, may be detected in the upper respiratory tract without causing symptoms. Conversely, upper airway samples may yield false-negative results in patients with lower respiratory tract involvement. Furthermore, the co-detection of bacterial and, less frequently, fungal pathogens is common. Nevertheless, respiratory viruses are now widely recognized as independent causes of severe disease, particularly in older adults and those with underlying comorbidities (especially the immunocompromised), and occasionally in previously healthy individuals. These infections can also exacerbate chronic conditions and increase the risk of secondary infections.

The objective of this narrative review is to provide a comprehensive synthesis of the current evidence on the management of immunocompetent adults admitted to intensive care units (ICUs) for community-acquired severe acute respiratory infections (SARIs) caused by respiratory viruses (excluding SARS-CoV-2). The review specifically focuses on viral pathogens transmitted through the respiratory route and on immunocompetent adults. Herpesviruses will not be discussed, as they are not typical respiratory pathogens, are predominantly detected in nosocomial settings, and are primarily associated with viral reactivation and relative immunosuppression in mechanically ventilated patients, rather than with true respiratory acquisition.

### Literature Search Strategy

This review is based on a comprehensive literature search conducted in PubMed and Google Scholar, focusing on publications from the period following the widespread implementation of PCR testing. For each respiratory virus addressed, all relevant studies were reviewed from the time PCR testing became routinely available, with earlier publications included when essential for clinical or historical context. Search strings combined MeSH terms and free-text keywords using Boolean operators, for example: (“lower respiratory tract infection” OR “pneumonia” OR “viral pneumonia”) AND (“influenza” OR “RSV” OR “respiratory syncytial virus” OR “human metapneumovirus” OR “rhinovirus” OR “adenovirus” OR “parainfluenza” OR “coronavirus”). The search was limited to English-language literature. Case reports were excluded, except for those concerning human bocavirus, due to the paucity of available clinical data. Recent and relevant peer-reviewed studies—including observational cohorts, randomized controlled trials, and meta-analyses—were prioritized. Reference lists of key articles were manually screened to ensure completeness. A total of 2897 records were initially identified through title screening. A subset was selected for abstract review, followed by the full-text evaluation of studies deemed relevant to the scope of this review.

## 2. Impact of Molecular Testing in Discovering Viruses in Lower Respiratory Tract Infections

The introduction of multiplex PCR (mPCR) after 2007, allowing the simultaneous detection of a broad spectrum of respiratory viruses, has profoundly transformed the epidemiological understanding of lower respiratory tract infections (LRTIs). Available platforms demonstrate a positive predictive agreement exceeding 95% when compared with gold-standard reference methods [1,2]. However, the clinical relevance of viral detection remains uncertain, ranging from true pathogenicity to incidental colonization or transient respiratory carriage. This variability likely depends on the specific virus involved, the host immune status, the timing of the specimen collection, and the anatomical sampling site.

While nasopharyngeal PCR remains the standard of care (SOC) for viral detection in most clinical settings, its relevance for diagnosing LRTIs is debated. Studies have reported both underdiagnosis—when upper airway samples yield false-negative results despite lower tract involvement—and overdiagnosis, particularly in the context of asymptomatic viral carriage or prolonged viral shedding [3]. Evidence from longitudinal nasopharyngeal sampling in U.S. households has demonstrated that a viral presence in the upper respiratory tract is common. In 108 individuals from 26 families sampled weekly, 783 viruses were recovered from nasopharyngeal PCR tests and were associated with respiratory symptoms in only 56% of cases [4]. A prolonged viral detection lasting more than 4 weeks was common for bocavirus and rhinovirus, likely reflecting either prolonged excretion or reinfections. These discrepancies highlight the limitations of relying solely on upper respiratory tract specimens for the diagnosis of severe viral pneumonia.

The use of mPCR assays that integrate both bacterial and viral targets may further influence the reported prevalence of viral infections. Among commercially available tools, the FilmArray Pneumonia Panel (FA-PP, bioMérieux™, Marcy l’Etoile, France) is currently the only mPCR platform that includes eight viral targets. Although increasing evidence supports the role of bacterial–viral co-infections in exacerbating pneumonia severity, the clinical utility of the FA-PP for their detection remains incompletely established [5,6,7,8]. The interpretation of viral epidemiology by the FA-PP is challenging due to substantial heterogeneity in study populations. Some studies focus exclusively on FA-PP-positive bacterial samples, which inherently biases the viral detection towards bacterial–viral co-infections [9,10,11]. Furthermore, studies often confound community- and hospital-acquired LRTIs or are limited to cohorts of SARS-CoV-2 infections, limiting conclusions about the broader role of viral detection [11,12]. Across published studies, viral detection rates using the FA-PP range from 15% to 51%, while bacterial–viral co-infections are identified in 10% to 38% of cases, depending on the population and pneumonia subtype [13,14,15,16,17,18,19,20,21,22]. In one study, the FA-PP identified nearly twice as many viral pathogens as the SOC testing, likely due to the broader scope of sputum testing compared to nasopharyngeal sampling [18]. However, in 95% of cases where the FA-PP was the only method used to detect a virus, no SOC test was performed, limiting direct comparisons and suggesting the underuse of viral diagnostics.

Collectively, these findings suggest that viral detection using mPCR pneumonia panels may provide useful clinical insight, particularly in critically ill patients. Nevertheless, the true impact of such diagnostics on clinical decision-making and patient outcomes remains to be clearly established. Further research is needed to clarify their role in pneumonia management. Table 1 summarizes the key features of currently available commercial respiratory mPCR kits.

## 3. Physiopathology of Respiratory Viral Infections

### 3.1. Epithelial Tropism and Viral-Induced Cell Death

Respiratory viruses, including influenza, RSV, and hMPV, demonstrate a marked tropism for the respiratory epithelium, infecting both the upper and lower respiratory tract. Influenza primarily targets ciliated epithelial cells in the upper airways and extends to secretory cells and type I and II alveolar epithelial cells in the lower respiratory tract [23,24]. Emerging evidence indicates that α2-6 sialic acids, predominantly expressed in the upper respiratory tract, are also present in respiratory bronchioles. This enables the viral spread to the lower airways, exacerbating epithelial damage and contributing to alveolar collapse [25,26]. Similarly, RSV and hMPV compromise the epithelial barrier by disrupting tight junctions and impairing ciliary function, thereby facilitating viral dissemination throughout the respiratory tree [27,28]. Adenoviruses, although less common, exhibit a similar capacity to infect alveolar structures and cause severe pulmonary injury, even in immunocompetent hosts [29,30]. Virus-induced cell death, including apoptosis and necrosis, exacerbates this epithelial damage [31,32]. For example, influenza and RSV trigger mitochondrial dysfunction and caspase activation, which impair repair mechanisms and compromise respiratory integrity [33,34]. Advanced experimental models, such as organoids and transcriptomics, further support the ability of these viruses to infect and damage alveolar epithelial cells, highlighting their capacity to disrupt the respiratory barrier [27,35]. Collectively, these findings underscore that the pathogenesis of these viruses involves the entire respiratory tree, explaining their central role in severe LRTIs and acute respiratory failure (ARF).

### 3.2. Immune Dysregulation and Inflammation-Mediated Injury

Respiratory viruses trigger immune responses that are essential for viral clearance but can also drive significant immune-mediated lung injuries [36]. Influenza infection induces a strong activation of macrophages and dendritic cells, resulting in the release of cytokines such as TNF-α, IL-6, and type I interferons. While these mediators are critical for recruiting immune cells and priming adaptive immunity, the excessive release may drive a “cytokine storm”, amplifying inflammation and causing diffuse alveolar damage and acute respiratory distress syndrome [37]. Similarly, RSV induces toll-like receptor activation on epithelial cells, leading to the production of chemokines, like IL-8, which recruit neutrophils and monocytes [27,38]. Although essential for pathogen clearance, these immune cells release reactive oxygen species and proteolytic enzymes that can exacerbate the epithelial injury. Respiratory viruses also alter the function of alveolar macrophages and CD8+ T cells. Macrophages, while pivotal for viral clearance, may adopt a pro-inflammatory phenotype, releasing TNF-α and IFN-γ. This overactivation contributes to epithelial disruption and lung injury. Similarly, CD8+ T cells eliminate infected epithelial cells but, when excessively activated, can cause tissue destruction and fibrosis [37]. Moreover, epithelial barrier disruption and impaired mucociliary clearance facilitate bacterial superinfections, particularly with *Streptococcus pneumoniae* and *Staphylococcus aureus*. This process is compounded by IFN-γ, which suppresses the alveolar macrophage-mediated bacterial clearance, further worsening lung injuries [39,40,41].

### 3.3. Histopathological Evidence from Human Studies

**Figure 1:**
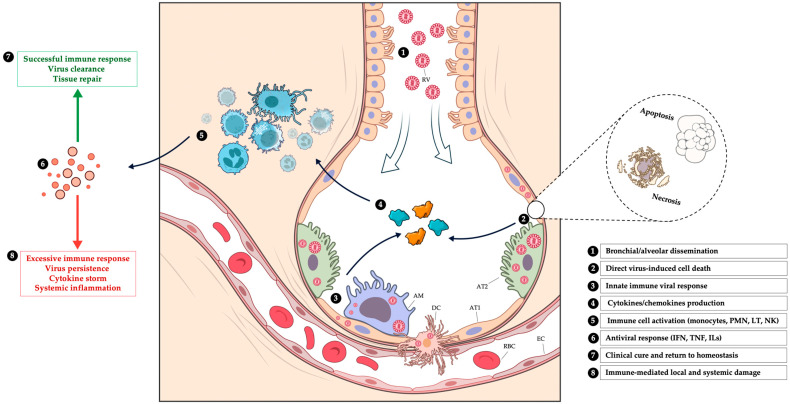
Pathophysiology of lung damage induced by respiratory viruses. AM: alveolar macrophages, AT1: alveolar type 1 cell, AT2: alveolar type 2 cell, DC: dendritic cell, EC: endothelial cell, IFN: interferon, ILs: interleukines, LT: T cell, NK: natural killer cell, PMN: polymorphonuclear leukocyte, RBC: red blood cell, and RV: respiratory virus. Illustrations from NIAID NIH BIOART Source Public Domain (bioart.niaid.nih.gov).

Histopathological studies demonstrate that respiratory viruses, including influenza, RSV, hMPV, and adenoviruses, cause significant injury in the lower respiratory tract. Findings from autopsy and biopsy studies reveal necrotizing bronchitis and bronchiolitis, diffuse alveolar damage with hyaline membranes, alveolar edema, and inflammatory cell infiltration as hallmarks of severe influenza infection [42]. RSV shows similar lesions, with the sloughing of the bronchiolar epithelium, mucus plugging, and neutrophilic alveolitis, contributing to airway obstruction and alveolar inflammation [43]. Adenoviruses induce necrotizing bronchopneumonia and interstitial inflammation, with severe cases reported in immunocompetent patients [44]. Although histopathological data on hMPV are scarce, the available evidence supports its potential to provoke interstitial and alveolar inflammation [45]. Across these infections, epithelial disruption facilitates secondary bacterial infections, which further exacerbate alveolar damage. The physiopathology of the respiratory viral lung injury is illustrated in Figure 1.

## 4. Clinical Consequences of Respiratory Virus in Severe Acute Respiratory Failure in ICU

Severe respiratory viral infections are a frequent cause of ICU admissions, typically presenting as ARF with or without pneumonia. Non-pneumonic ARF is often linked to exacerbations of underlying comorbidities, particularly chronic obstructive pulmonary disease (COPD) and cardiovascular conditions [46].

While the association between respiratory viruses and COPD exacerbations is well established, emerging evidence underscores their role in triggering cardiovascular complications. In a cohort of 6248 adults aged ≥50 years hospitalized with laboratory-confirmed RSV infections, 22.4% experienced acute cardiac events, including acute heart failure (15.8%), acute ischemic heart disease (7.5%), and hypertensive crises (1.3%) [47]. Influenza has similarly been shown to markedly increase vascular risk, with one study reporting a nearly tenfold rise in myocardial infarction risk (IRR 9.80, 95% CI 2.37–40.5) and a twelvefold increase in stroke risk (IRR 12.3, 95% CI 5.48–27.7) during the first three days post-infection [48]. These findings highlight the capacity of respiratory viruses not only to cause direct pulmonary injury but also to precipitate systemic complications.

In addition to non-pneumonic presentations, respiratory viruses are well-recognized causes of community-acquired pneumonia (CAP), with viral etiologies identified in approximately 30–40% of cases [5,49,50,51]. These infections typically present with systemic and respiratory symptoms, including fever, cough, myalgia, anorexia, and headache, with a median incubation period ≤ 7 days [52]. Importantly, this clinical presentation is neither specific to viral etiologies nor sufficient to distinguish between viral and bacterial causes [5,53]. Co-infections with bacterial pathogens are reported in up to 25% of viral pneumonia cases and are associated with a more severe presentation, including higher rates of sepsis and mechanical ventilation [5,54]. Thus, bacterial sampling remains crucial to guide timely antimicrobial therapy even when a viral pathogen is identified. These observations underscore the clinical heterogeneity of respiratory viral infections in ICU patients and the necessity of an integrated diagnostic approach to identify co-infections and systemic complications. Selected clinical vignettes are presented in Table 2.

## 5. Outcomes

Despite the clinical burden of respiratory viral infections, population-based epidemiological studies comparing outcomes remain scarce. Bajema et al. studied a retrospective cohort of 219,577 patients with a median age of 66 years from electronic health record data of non-hospitalized U.S. veterans who underwent same-day testing for SARS-CoV-2, influenza, and RSV during the autumn–winter season in 2023 and 2024 and had a single positive result (SARS-CoV-2 63%, RSV 11%, and influenzae 26%) [64]. The 30-day risk of hospitalization was similar for COVID-19 (16.2%) and influenza (16.3%), but lower for RSV (14.3%). The ICU admission rate was slightly higher for SARS-CoV-2 patients (3%) compared to RSV (1.8%) and influenza (1.5%). The 90-day risk of death was similar between the three (SARS-CoV-2: 1.8%, RSV: 1.4%, and influenzae: 1.3%).

In ICU settings, viral CAP exhibits a severity profile comparable to bacterial pneumonia, with median ventilation durations ranging from 7 to 10 days and mortality rates between 10% and 30% [5,50,65,66]. However, not all respiratory viruses carry the same prognostic weight. Among them, influenza and RSV have been consistently associated with severe outcomes in hospitalized and ICU populations. Grangier et al. reported comparable ICU lengths of stay for RSV and influenza (6–7 days), with mortality rates of 29% and 25%, respectively [65]. Similarly, Coussement et al. found no significant difference in ICU mortality between RSV (23.9%) and influenza (25.6%) [67]. hMPV, though less frequently identified, is another pathogen associated with severe disease, with ICU admission rates from 10% to 30% and early mortality between 3% and 10% [61,68,69].

By contrast, the pathogenic potential of other respiratory viruses in immunocompetent adults remains less clear. Adenoviruses, while capable of causing severe disease in immunocompromised individuals, rarely lead to severe outcomes in healthy adults. For instance, in one study involving military trainees, only 4.7% of adenovirus infections required ICU admission, and severe complications, such as ARDS, were isolated events [50,70,71]. Parainfluenza viruses, though frequently detected, are typically associated with mild disease in immunocompetent individuals, with ICU mortality rates of approximately 3% and a minimal need for mechanical ventilation [72,73]. Data on rhinovirus are more equivocal. Retrospective ICU studies estimate a mortality rate of around 30%, but these numbers are frequently confounded by co-infections or underlying conditions [74,75]. Seasonal coronaviruses, such as OC43, NL63, and 229E, are rarely implicated in severe pneumonia among immunocompetent adults, and their role as primary pathogens remains unclear [50,76,77]. Human bocavirus exhibits a similarly doubtful pathogenicity, with only sporadic reports of ARDS or ICU admission in adults [78,79]. 

Beyond viral etiology, bacterial co-infections represent a critical determinant of outcomes in ICU patients. Such co-infections have been reported in up to 25% of patients, depending on patient characteristics and the extent of bacterial sampling [5,46,50,51,80,81,82,83]. In ICU settings, Voiriot et al. showed that patients with mixed infections had higher mechanical ventilation rates and mortality (28.9%) compared with patients with isolated bacterial (13%) or viral (11.3%) infections [5]. These findings are supported by a meta-analysis showing a twofold increase in the mortality risk associated with mixed infections (OR 2.1, 95% CI 1.32–3.31) [51]. Furthermore, a database study involving 15,906 patients with viral respiratory infections revealed that mixed infections were associated with a threefold increase in ICU admissions (OR 2.9, 95% CI 2.3–3.6) and 30-day mortality (OR 2.6, 95% CI 1.9–3.7) [82].

Cardiovascular complications are another key factor worsening outcomes. In a large cohort of RSV-related hospitalizations, acute cardiac events were significantly associated with higher ICU admission rates (25.8% vs. 16.5%; ARR 1.54, 95% CI 1.23–1.93) and in-hospital mortality (8.1% vs. 4.0%; ARR 1.77, 95% CI 1.36–2.31) [47]. These findings underscore the critical importance of the early recognition and targeted management of cardiovascular complications in ICU patients with viral respiratory infections. Importantly, the role of respiratory viruses in precipitating COPD exacerbations has been extensively reviewed elsewhere. Clinical outcomes by virus types are summarized in Table 3.

### Population at Risk of Severe Outcome

Among immunocompetent patients, an older age and pre-existing comorbidities—especially cardiopulmonary disease—are the most consistent predictors of adverse outcomes [92,93]. RSV hospitalization rates increase significantly with age, reaching 136.9–255.6 per 100,000 in individuals aged ≥65 years, with even higher rates observed in those with COPD, coronary artery disease, or heart failure [94]. Beyond acute complications, severe infections in older adults often lead to prolonged functional decline; for instance, 33% of RSV-hospitalized patients exhibit persistent impairments six months post-discharge [95]. Similarly, influenza disproportionately affects patients with pre-existing comorbidities, substantially increasing risks of hospitalization, ICU admission, and mortality [93]. These findings underscore the importance of targeted prevention and management strategies for high-risk populations, particularly older adults and individuals with cardiopulmonary diseases, to reduce both the immediate and long-term impacts of severe respiratory viral infections. Vaccines effective against RSV and influenza are cornerstones of the prevention strategy in these populations [96].

## 6. Treatment of Severe Respiratory Viral Infections

### 6.1. Noninvasive Respiratory Support in Viral Pneumonia

The COVID-19 pandemic re-established noninvasive respiratory support as a central component of acute respiratory failure management. Observational data from large cohorts initially suggested potential benefits of alternatives to standard oxygen therapy. In the COVID-19-ICU cohort (*n* = 4754), high-flow nasal cannula (HFNC) was associated with a reduced risk of oxygenation failure—defined as intubation or death without intubation—compared to standard oxygen (adjusted OR 0.60; 95% CI 0.36–0.99), whereas noninvasive ventilation was associated with an increased 90-day mortality (adjusted OR 2.75; 95% CI 1.79–4.21) [97]. However, these findings were not confirmed in the COVIDICUS randomized trial (*n* = 546), which compared HFNC, CPAP, and standard oxygen in ICU patients with COVID-19. The study found no significant differences in 28-day intubation rates between HFNC and standard oxygen (HR 1.04; 95% CI 0.69–1.55), or between CPAP and standard oxygen (HR 1.08; 95% CI 0.71–1.63), challenging the assumption that advanced noninvasive strategies are superior in this setting [98].

There are currently no randomized data specifically addressing non-COVID-19 viral pneumonia. In this context, the evidence must be extrapolated from broader populations with acute hypoxemic respiratory failure. A 2024 meta-analysis of 63 studies (*n* = 10,230) evaluated HFNC versus conventional oxygen therapy across various etiologies, including COVID-19 (*n* = 3782) and non-COVID-19 pneumonia (*n* = 1583). HFNC was associated with a reduced escalation to invasive ventilation (RR 0.85; 95% CI 0.76–0.95) and to noninvasive ventilation (RR 0.70; 95% CI 0.50–0.98) but had no effect on hospital mortality (RR 1.08; 95% CI 0.93–1.26). Although the population included some patients with non-COVID-19 pneumonia, no prespecified subgroup analysis was conducted.

Taken together, while HFNC appears safe and may reduce the need for intubation in selected patients, the current evidence does not support the preferential use of any specific noninvasive strategy in non-COVID-19 viral pneumonia. Outside of well-established indications—such as noninvasive ventilation in acute exacerbations of COPD or cardiogenic pulmonary edema—the choice of respiratory support remains empirical. Dedicated randomized trials are needed to evaluate ventilatory strategies in patients with confirmed viral pneumonia.

### 6.2. Antiviral Therapy

For severe influenza-associated pneumonia, neuraminidase inhibitors (NAIs), particularly oseltamivir, remain the cornerstone of treatment despite limited evidence in critically ill patients. Key questions persist regarding the effectiveness of antivirals on patient outcomes, the optimal duration of therapy (5 days vs. prolonged courses), and whether monotherapy or combination regimens offer superior benefits. A recent meta-analysis of eight randomized controlled trials in severe influenza reported no significant differences in mortality or ICU admissions compared to placebos, but oseltamivir and peramivir reduced hospital stays by 1.63 and 1.73 days, respectively [99]. Observational studies suggest that an extended oseltamivir therapy (≥10 days) may yield better outcomes in ICU settings, with a 6.2% absolute reduction in mortality (adjusted OR: 0.53, 95% CI: 0.40–0.69), although these findings await confirmation in randomized trials [100]. Combination regimens, such as oseltamivir–zanamivir, have failed to demonstrate an added efficacy and may increase adverse effects [101]. Similarly, novel agents, like baloxavir, targeting distinct viral replication pathways, have not shown superiority in hospitalized patients. The FLAGSTONE trial confirmed that baloxavir combined with NAIs offered no clinical advantage over NAI monotherapy in severe cases [102]. Ongoing studies, including the REMAP-CAP trial (http://clinicaltrials.gov: NCT02735707), are expected to provide more clarity on the optimal antiviral strategies for critically ill patients with influenza.

RSV treatment remains a significant challenge. Ribavirin, historically considered for RSV, has demonstrated an inconsistent efficacy and potential toxicity, limiting its role, particularly in immunocompetent patients [81,103]. While prophylactic monoclonal antibodies, such as palivizumab and nirsevimab, have been explored for infants and high-risk children, their therapeutic potential in adults remains unproven and requires further investigation. Emerging agents, like zelicapavir, are currently under evaluation for older adults with cardiopulmonary comorbidities, but robust trial data are still awaited. For other respiratory viruses, effective antiviral therapies are unavailable, making supportive care, such as oxygen supplementation, advanced ventilatory strategies, and monitoring for bacterial superinfections, the primary approach. Promising investigation agents targeting viral fusion proteins and replication pathways show potential but remain in early development. Table 4 summarizes therapeutic options available in cases of severe RVI.

### 6.3. Immunomodulation

The role of corticosteroids in viral pneumonia remains highly debated. The CAPE-COD trial demonstrated a mortality reduction with low-dose hydrocortisone in severe CAP, though subgroup analyses did not specifically address viral etiologies [114]. In influenza-associated pneumonia, observational studies and meta-analyses have consistently indicated that corticosteroid use is associated with an increased mortality (OR 3.90, 95% CI 2.31–6.60) and higher rates of hospital-acquired infections (OR 2.74, 95% CI 1.51–4.95) [115]. However, these findings must be interpreted cautiously, as the absence of high-quality RCTs and the potential for confounding by indications—given that corticosteroids are often used in the most critically ill patients—complicate definitive conclusions. Beyond influenza, data on corticosteroid use in other viral respiratory infections remain limited, and no randomized controlled trials currently provide clear guidance. A recent individual participant data meta-analysis by Smit et al., evaluating corticosteroid therapy in severe CAP, found no statistically significant heterogeneity in treatment effect based on microbiological etiology [116]. However, point estimates of mortality reduction consistently disfavored corticosteroids in viral infections: −2.6% (95% CI −7.1 to 1.5) for viral CAP, −4.0% (−9.4 to 1.0) for viral-only CAP, −3.6% (−11.5 to 4.2) for influenza, and −4.4% (−13.4 to 4.2) for influenza-only cases. These trends underscore the need for caution when considering corticosteroids in the management of viral pneumonia in the absence of robust pathogen-specific evidence.

Efforts to address these evidence gaps are ongoing. Trials such as REMAP-CAP (http://clinicaltrias.gov: NCT02735707) and RECOVERY (http://clinicaltrials.gov: NCT04381936) are investigating the efficacy of corticosteroids and other immunomodulatory agents, including tocilizumab and baricitinib, across various viral pneumonias. These trials are essential for establishing evidence-based guidelines and optimizing outcomes in critically ill patients. Until then, the decision to use immunomodulatory therapies should remain highly individualized, carefully balancing potential risks—such as delayed viral clearance and secondary infections—against plausible clinical benefits in selected cases.

## 7. Vaccination as a Key Strategy Against Severe Respiratory Viral Infections

While antiviral treatments have shown variable efficacy, vaccination remains the most effective strategy for preventing severe respiratory viral infections (RVIs), particularly in high-risk populations such as the elderly. Among common seasonal respiratory viruses, vaccines are currently available for influenza and RSV, in addition to SARS-CoV-2.

For influenza, vaccine effectiveness is largely dependent on the antigenic match with circulating strains during a given season [117]. The greatest benefit is observed in individuals aged ≥65 years. A systematic review by Demicheli et al. found that influenza vaccination reduced the risk of confirmed influenza from 6% to 2.4%, and likely decreased the incidence of influenza-like illness from 6% to 3.5% in this population [118]. Newall et al. estimated that in the United States, a 1% increase in the overall influenza vaccine uptake during the influenza season was associated with a 0.33 (95% CI: 0.20–0.47) per 100,000 population reduction in pneumonia- and influenza-related deaths [119]. Similar findings were reported in France between 2000 and 2009, where vaccination prevented 2000 deaths annually and had an estimated effectiveness of 35% against influenza-attributable mortality. In this population, approximately 2650 vaccinations were required to prevent one influenza-related death among older adults [120].

A newly approved RSV vaccine has recently become available for older adults. Real-world data from an epidemic season (October 2023–March 2024) demonstrated that in individuals aged ≥60 years, the RSV vaccine effectiveness was 77% (95% CI: 70–83) against RSV-associated emergency department visits, 80% (95% CI: 71–85) against RSV-related hospitalization, and 81% (95% CI: 52–92) against ICU admission or death [121].

Finally, ensuring a high vaccine coverage against respiratory viruses that are now rare due to near-eradication remains crucial, as illustrated by the recent resurgence of measles in the United States (228 cases as of 7 March 2025) [122,123]. Measles is highly contagious (12–18 secondary cases per infected individual) and potentially fatal [124]. The current recommended two-dose vaccination strategy is highly effective, providing 90–99% protection. In infected individuals, pneumonitis is frequent but usually mild in immunocompetent patients; however, ARF requiring ICU admission occurs in about 3% of the cases [125]. In critically ill patients, the disease is much more severe. A French ICU cohort from the 2009–2011 outbreak showed that measles primarily affected young patients (median age: 29.2 years) who had not received the full two-dose vaccine regimen. The disease progression was severe, with ARDS occurring in 9 of 36 patients and mortality in 5 of 36 cases [126]. These findings collectively underscore the central role of vaccination in mitigating the burden of severe RVIs—both from currently circulating pathogens and from those re-emerging due to lapses in immunization coverage. For measles in particular, maintaining high vaccination rates is essential, as severe complications can occur even in previously healthy adults, and no specific antiviral treatment is available.

## 8. Future Risks

### 8.1. Post-Pandemic Effects

The COVID-19 pandemic has profoundly reshaped the epidemiology of respiratory pathogens. Non-pharmaceutical interventions, such as lockdowns, mask mandates, and travel restrictions, led to an unprecedented decline in pathogen circulation. Influenza transmission, for instance, was reduced by over 95%, significantly disrupting global patterns of spread [127,128]. While these measures reduced short-term transmission, they also limited natural immunity development—a phenomenon referred to as “immunity debt”. Interruptions in vaccination campaigns and a reduced vaccine uptake further weakened the herd immunity, heightening the risk of severe post-pandemic outbreaks.

The case of influenza A(H3N2) illustrates the cascading consequences of these disruptions. In Australia, genetic analyses revealed that dominant H3N2 strains in 2022 (subclade 3C.2a1b.2a.2) were introduced via international travel after restrictions were lifted [129]. The antigenic drift between successive strains was linked to more intense epidemics, marked by higher transmission rates, increased adult cases, and H3N2 dominance [127,129,130]. Such examples demonstrate the enduring consequences of pandemic-related disruptions, emphasizing the importance of continuous surveillance, robust vaccination efforts, and targeted public health measures to address these evolving risks.

### 8.2. Emerging Threats

Beyond the established endemic respiratory viruses, emerging viral threats pose an increasing challenge to global health, driven by environmental changes, globalization, and evolving pathogen characteristics. The SARS, MERS, and COVID-19 pandemics underscore the potential for novel pathogens to cause severe outbreaks, with high mortality rates and a significant global impact [131,132]. Influenza remains a persistent concern, particularly the emergence of highly pathogenic avian strains with pandemic potential [63,133]. A(H5N1) virus spread from east Asia to west Asia and Africa is the most common. It was associated with a hospitalization rate of more than 90% and a case fatality rate of more than 50% [134]. Although few clustering cases have been reported, human to human transmission is unlikely. Beyond these known threats, arboviruses such as dengue and chikungunya are expanding geographically due to climate change and the proliferation of mosquito vectors [135,136]. This geographical shift poses a growing risk of outbreaks in previously unaffected regions, including parts of Europe. The resurgence of measles due to a decrease in vaccination coverage should also be kept in mind. These evolving dynamics highlight the urgent need for robust surveillance systems, cross-border collaboration, and innovative research to anticipate, detect, and mitigate the impact of respiratory viral emergencies. As masking is effective in reducing contamination, the situations where it should be recommended or mandated as well as the optimal filtration characteristics should be better defined [137].

## 9. Conclusions

Severe respiratory viral infections remain a major cause of ICU admissions, particularly in older adults and those with comorbidities. The widespread use of molecular diagnostics has improved viral detection, yet its clinical relevance, particularly in differentiating colonization from infection, remains debated. Influenza and Pneumoviridae (RSV, hMPV) are the most severe pathogens, often complicated by bacterial co-infections that worsen outcomes. While neuraminidase inhibitors are standard for severe influenza, effective antiviral options for other respiratory viruses are lacking. Future research should focus on optimizing antiviral strategies, refining the role of immunomodulation, and improving the early identification of high-risk patients to enhance clinical outcomes.

## Figures and Tables

**Table 1:** Commercially available respiratory mPCR kits in 2025: key features and viral targets.

	Xtag Respiratory Viral Panel FastV2	Respifinder Smart 22 Fast	Allplex Respiratory Panel Assays	Filmarray Respiratory Plus 2,1 Panel	ePlex Respiratory Pathogen 2 Panel	QIAstatDx Respiratory Panel 2
	Luminex	Pathofinder	Seegene	Biofire	GenMark Dx	Qiagen
PCR	Semi quantitative	Final	Real time	Final	Final	Real time
Turn-around time	4 h	6 h	4 h 30 min	45 min	70 min	70 min
Targets						
**Influenzae A and B**	X	X	X	X	X	X
**Respiratory syncytial virus**	X	X	X	X	X	X
**Rhinovirus/enterovirus**	X	X	X	X	X	X
**Human metapneumovirus**	X	X	X	X	X	X
**Human coronaviruses**	X	X	X	X	X	X
**Parainfluenza virus**	X	X	X	X	X	X
**Adenovirus**	X	X	X	X	X	X
**Bocavirus**	X	X	X		X	X
**Mers-Cov**				X	X	
**SARS-CoV-2**				X	X	X

**Table 2:** Clinical vignettes associated with major respiratory viruses.

Virus	Clinical Vignettes/Specific Data	Precautions/Prevention
***Common respiratory viruses***		
**Influenza A and influenza B** [55,56]	Short incubation (mean 2 days); viral shedding from 2 days prior to symptoms and peaks within 2–3 days afterwards. The most common cause of ICU admission either with pneumonia or with acute exacerbation of chronic or respiratory diseases. May be associated with acute myocardial infarction, myocarditis, rhabdomyolysis, acute renal failure, encephalopathy/encephalitis, and other non-pulmonary complications.	Droplet /Yearly vaccine
**SARS-CoV-2** [57,58,59]	New variants are associated with high transmissibility, milder diseases, and immune escape (JN.1 is the most common sublineage; KP 3.1.1 is rapidly growing). Case fatality rate is 1.9% higher in the elderly and low- and middle-income countries. Severe forms are observed in immunocompetent patients with comorbid conditions (chronic diseases, obesity, and elderly). Compared to historical variants, higher rate of cases in vaccinated people (but delay since the last dose) and shorter delay from first symptom to ICU admission (5 days).	Droplet + Airborne /Vaccine
**Respiratory syncytial virus** [46,60]	Winter season. Elderly patients with chronic respiratory or cardiac diseases. Immunodepression in one-third of the cases of poor prognosis. Commonly associated with exacerbation of chronic respiratory or cardiac insufficiency. Bronchospasm is common. New vaccines offer about 90% protection to adults over 65.	Contact /Vaccine
**Human metapneumovirus** [61]	Elderly patients with chronic respiratory or cardiac diseases. Hospital admission after a median delay of 3 days of symptoms. Half of patients present with pneumonia (interstitial). Immunodepression in one-third of the ICU cases.	Contact
**Adenoviruses** [62]	Usually mild symptoms, keratoconjunctivitis, and gastro-intestinal symptoms. Severe forms are rare but observed in young and middle-aged adults (median age 40 yo). ARDS is common. Some cases are associated with hepatitis. Rare cases of myocarditis, cardiomyopathy, pancreatitis, encephalitis, meningitis, and mononucleosis-like syndromes.	Droplet + contact
**Picornaviruses** **(rhinovirus and enterovirus)**	Frequently detected in critically ill patients with severe acute respiratory infection. Excretion > 2 months are common. Questionable impact on respiratory insufficiency and prognosis in immunocompetent adults.	Droplet
**Human coronaviruses** **(229E, NL63, OC43, and HKU1)** **Parainfluenza (1–4)**	Year-long transmissibility. May cause severe illness in the elderly, persons with comorbidities including immunosuppression. Parainfluenza: usually mild upper respiratory diseases, cases of laryngotracheobronchitis (croup) and bronchiolitis.	Contact
***Uncommon and emerging viruses***		
**Avian influenza A/H5N1, A/H5N6, A/H7N9, and other subtypes** [63]	Residence in or travel to Southeast and East Asia. Exposure to poultry or visits to poultry markets. Severe ARDS.	Airborne + contact
**MERS-CoV**	Severe pneumonia, gastro-intestinal symptoms. Residence in or travel to the Arabian Peninsula. Exposure to dromedary camel (in endemic areas). Nosocomial transmission risk to other patients and to healthcare workers.	Airborne + contact
**Measles**	Incomplete vaccination. Characteristic cutaneous rash. Progressive giant cell pneumonia.	Airborne /Vaccine
**Hantaviruses** **(e.g., Sin Nombre and Andes)**	Residence in or travel to affected areas of North, Central, or South America. Exposure to rodent excretions.	Standard

Abbreviations: ARDS, acute respiratory distress syndrome. Isolation precautions: adapted from https://www.cdc.gov/infection-control/hcp/viral-respiratory-prevention/ (accessed on 24 March 2025). *NB: For all respiratory viruses: (1) Take measures to limit crowding in communal spaces, such as scheduling appointments to limit the number of patients in waiting rooms or treatment areas. (2) Encourage people with symptoms of respiratory infection to sit away from other patients. If possible, facilities may wish to place these people in a separate room while they are waiting for care. (3) During periods of increased community respiratory virus activity that results in a surge in visits, facilities could consider setting up triage stations that facilitate the rapid screening of patients for signs and symptoms of respiratory infection and separation from other patients.*

**Table 3:** Prognosis of severe viral respiratory infections in immunocompetent patients.

	ICU Admission in Hospitalized Patients (%)	During ICU Stay			
		Mechanical Ventilation (%)	Bacterial Co-Infection (%)	ARDS (%)	Mortality (%)
**Seasonal influenza** [56,84,85,86,87]	15–20	30–65	35	25–50	15–25
**Respiratory syncytial virus** [46,60,67,81]	15–20	30–35	25–35	15–20	10–15
**Human metapneumovirus** [61,68,69]	5–10	40–50	20	10–25	20
**Rhinovirus** [75,88,89]	15–20	50	30	?	30
Parainfluenza viruses * [73]	25	?	30	?	20–25
Adenovirus * [70]	5	40–50	?	10–20	0–5
Seasonal coronoaviruses * [90,91]	15–30	0–7	20–30	0–3	?
**Bocavirus** [78] *	?	Case reports	Case reports	Case reports	Case reports

Abbreviations: ARDS, acute respiratory distress syndrome. ? Missing data indicate insufficient evidence or lack of reported cases. * Data are derived from small sample sizes and should be interpreted with caution.

**Table 4:** Available antiviral therapies for viral pneumonia.

Treatment	Outcome	Comment
**Influenza**		
**Oseltamivir** [99,104,105,106] (oral)	***Outpatient population*** No reduction in hospitalization risk in the general population (RR, 0.79; 95% CI, 0.48–1.29) No reduction in hospitalization risk in high-risk patients (RR, 0.65; 0.33–1.28) No reduction in hospitalization risk in patients >65 years (RR, 1.01; 95% CI, 0.21–4.90) ***Hospitalized population*** Modest reduction in hospital stay duration (mean difference −1.63 days, 95% CI −2.81 to −0.45) No impact on ICU admission No impact on mortality	No specific serious adverse events Reported resistance: ~1% of strains globally
**Peramivir** [99,107,108] (IV)	***Outpatient population (compared to oseltamivir)*** No difference in time to alleviation of influenza symptoms ***Hospitalized population (compared to oseltamivir)*** No difference in hospital stay duration No impact on ICU admission No impact on mortality	No specific serious adverse events Rare resistance Second-line therapy: For oseltamivir resistance or when oral administration is not possible
**Zanamivir** [109] (inhaled, IV)	***Hospitalized population (compared to oseltamivir)*** No difference in time to alleviation of influenza symptoms No impact on ICU admission No impact on mortality	No specific serious adverse events Rare resistance Second-line therapy: For oseltamivir resistance or when oral administration is not possible
**Baloxavir** [102,110,111] (oral)	***Outpatient population (compared to oseltamivir)*** No difference in time to alleviation of influenza symptoms ***Hospitalized population: baloxavir + oseltamivir compared to oseltamivir alone*** No clinical benefit from adding baloxavir	Second-line therapy: For oseltamivir resistance (no cross-resistance with neuraminidase inhibitors)?
**Respiratory syncytial virus**		
**Ribavirin** [112] (oral, inhaled, IV)	Reduced mortality in LRTIs in patients with hematologic malignancies or hematopoietic stem cell transplants (aOR 0.19 [0.07, 0.51]) No indication for immunocompetent patients	Adverse events: nephrotoxicity, anemia, and rash
**Adenovirus**		
**Cidofovir** [113] (IV).	Weak evidence (mainly case reports in pediatric patients) suggesting potential effect on adenovirus clearance in immunocompromised patients	Adverse events: nephrotoxicity and leukopenia

## Abbreviations

The following abbreviations are used in this manuscript:

ARDS	acute respiratory distress syndrome
ARF	acute respiratory failure
CAP	community-acquired pneumonia
CDC	Centers for Disease Control and Prevention
CI	confidence interval
COPD	chronic obstructive pulmonary disease
COVID-19	Coronavirus Disease 2019
FA-PP	Film-Array Pneumonia Panel
HFNC	high-flow nasal cannula
hMPV	human metapneumovirus
ICU	intensive care unit
IFN	Interferon
IRR	incidence rate ratio
LRTI	lower respiratory tract infection
MERS	Middle East Respiratory Syndrome
mPCR	multiplex PCR
NAI	neuraminidase inhibitor
NPI	non-pharmaceutical intervention
OR	odds ratio
PCR	Polymerase Chain Reaction
RSV	respiratory syncytial virus
RVI	respiratory viral infection
SARI	severe acute respiratory infection
SARS	Severe Acute Respiratory Syndrome
SARS-CoV-2	Severe Acute Respiratory Syndrome Coronavirus 2
SOC	standard of care
TNF	tumor necrosis factor
